# Ionic liquids as electrolytes in aluminum electrolysis

**DOI:** 10.3389/fchem.2022.1014893

**Published:** 2022-11-03

**Authors:** Junshi Wu, Peng Xie, Wenbin Hao, Dong Lu, Ye Qi, Yongli Mi

**Affiliations:** ^1^ Department of Engineering Materials and Reliability, HKUST Fok Ying Tung Research Institute, Guangzhou, China; ^2^ Department of Chemical and Biological Engineering, Hong Kong University of Science and Technology, Kowloon, Hong Kong SAR, China; ^3^ Department of Chemical Engineering, School of Chemical Engineering and Technology, Xi’an Jiaotong University, Xi’an, China; ^4^ Department of Innovation, Policy and Entrepreneurship Thrust, Hong Kong University of Science and Technology (Guangzhou), Guangzhou, China

**Keywords:** ionic liquids, electrolytes, aluminum electrolysis, energy saving and emission reduction, environmentally friendly

## Abstract

Herein, the characteristics, research progress, and application prospects of ionic liquid-based electrolytic aluminum deposition are reviewed and analyzed in comparison with the Hall–Héroult method. The reaction conditions and production procedures of this material are discussed alongside the problems ionic liquids face in the electrolytic aluminum industry. Ionic liquid-based electrolytic aluminum deposition realizes the electrolytic aluminum reaction at low temperatures, achieving a reaction energy consumption close to the theoretical minimum value. The reaction also avoids harmful CO_2_ or HF emissions, demonstrating a green and environmental-friendly approach to the production of electrolytic aluminum. In the future, in-depth work on the implementation of ionic liquid electrolytes should be carried out, establishing the necessary technical criteria and laying the foundation for the integration of this approach.

## 1 Introduction

Aluminum, a silvery white light metal, is the most abundant metal in the earth’s crust. In recent years, aluminum has become one of the most widely used metals in the world due to its excellent properties, including its high electrical conductivity, high thermal conductivity, and light weight. The gradual industrial use of electrolytic aluminum by China since 1953 has led to the nation becoming a world leader in the industry. It is reported that the electrolytic aluminum output of China reached 44.9 million tons in 2017, accounting for more than 50% of the total production of primary aluminum in the global electrolytic aluminum industry.

China produced 38.5 billion tons of aluminum in 2021 (National Bureau of Statistics, 2022) and consumes roughly 519.79 billion kW·h of electricity in the production of electrolytic aluminum. However, the actual energy utilization rate of electrolytic aluminum is still less than 50%, with about half of the energy used lost to the environment due to the low energy efficiency of the methods used, namely the Hall–Héroult method. In addition, the high operating temperatures of this method induce more side reactions that result in severe environmental issues. Specifically, waste gases (namely CO, CO_2_, and HF) are generated at such a high operating temperature, some of which are toxic and severely harmful to human health. Although the exhaust emissions of electrolytic aluminum plants can generally meet the permitted environmental emission standards, they still far exceed the environmental carrying capacity of the production areas due to the high concentration of production plants situated in these areas. The enormous amount of exhaust emissions not only severely restricts the sustainable development of aluminum enterprises but also imposes a heavy burden on the local environment. Therefore, there is an urgent need to develop more environmentally friendly methods with higher energy efficiency to produce aluminum, especially at low temperatures or even room temperature (RT), which can significantly reduce energy consumption in the production of electrolytic aluminum.

In recent years, in order to reduce the electrolysis temperature and the harmful gases generated in the electrolysis of aluminum, low-temperature electrolytic aluminum produced using ionic liquids as the electrolyte has gradually gained attention. The process has the advantages of a reaction temperature below 150°C, an energy consumption below 10 kW h/kg, and no harmful gases or greenhouse gases generated during the reaction process. Thus, it presents itself as a disruptive technology compared to the traditional Hall–Héroult method that can also be applied to the preparation of high-purity aluminum and nano-aluminum as well as the research and application of aluminum-ion batteries.

## 2 The Hall–Héroult method

### 2.1 Overview

**FIGURE 1:**
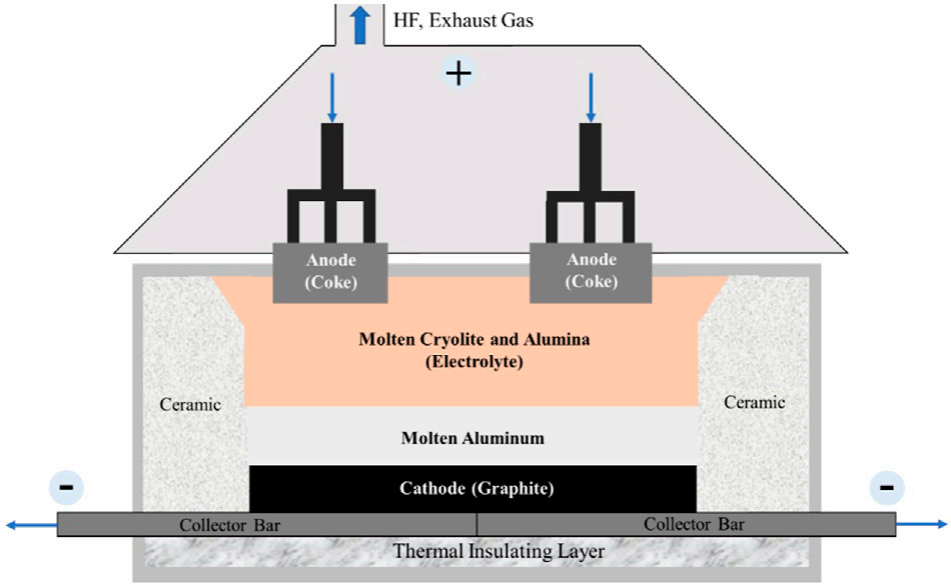
Structure of electrolytic cell used in the Hall–Héroult method (Li J. et al., 2011).

**FIGURE 2:**
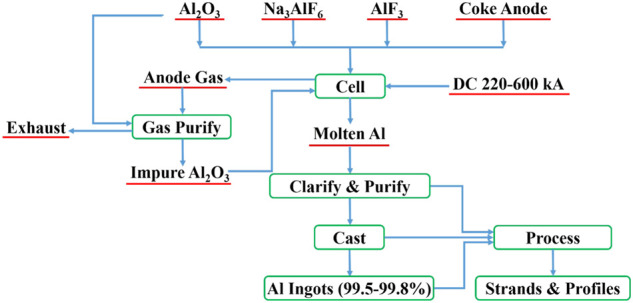
Flowchart of the Hall–Héroult aluminum production flowchart.

The electrolytic aluminum industry is the largest electrochemical industry in the world. Currently, only the Hall–Héroult method, namely cryolite-alumina molten salt electrolysis, is widely used in the large-scale modern industrial production of metal aluminum. The structure of the electrolytic cell used in this method is shown in Figure 1. In the Hall–Héroult method, electrolytic aluminum is produced using an electrolytic cell with coke as the anode and graphite as the cathode. Alumina is dissolved in the fused cryolite to form the molten electrolyte at 950–970°C. The reaction forming electrolytic aluminum occurs when a high-voltage direct current is applied to the cell. The anode product is mainly CO_2_ and CO and the cathode product is molten aluminum. The resultant molten aluminum is pumped out of the cell tank through a vacuum lifting system, clarified and purified in the holding furnace, and finally sent to the foundry workshop to cast into ingots or directly process into strands, profiles, *etc.* (Figure 2).

After over 100 years of improvement, the current Hall–Héroult method now has the advantages of low investment, simple facility, and short production period. However, the operating temperature of the Hall–Héroult process is usually more than 950°C, which results in extremely high energy consumption (13–15 kW h/kg Al) and low energy efficiency (≤50%).

### 2.2 Reducing energy consumption

Theoretically, reducing the working voltage in the Hall–Héroult method by 0.1 V can save about 320 kW h in the production of one ton of aluminum, while decreasing the electrolysis temperature by 10°C can save about 140 kW h (Xu and Li, 2010; Liu, 2013). Therefore, considerable efforts have been devoted to reducing the working voltage or decreasing the operating temperature to reduce the energy consumption of the Hall–Héroult method. Liang et al. developed a “static flow” electrolytic cell for aluminum electrolysis by optimizing the structure of the electrolytic cell to achieve long-term, efficient, and stable operation (China Industrial Economic Network, 2012). The working voltage of the electrolytic cell was reduced by 0.336 V and the operating temperature decreased by 23.5°C, resulting in 1,377 kW h energy saved per ton of aluminum produced (a total reduction of 10.7%). Many studies on saving energy in the high-temperature production of electrolytic aluminum have been carried out through the optimization of the electrolyte and the structure of the electrolytic cell (Li et al., 2006; Ren et al., 2007). However, due to the strong magnetic field in the electrolytic cell during the production of electrolytic aluminum, it is difficult to further reduce the working voltage (Li et al., 2011b). Reducing the high temperatures (≥900°C) used in electrolytic aluminum production is even more challenging due to the difficulties in finding a suitable electrolyte with both low melting temperature and good solubility to alumina (Hou and Qiu, 2011). Therefore, there are few ways to further improve the efficiency of the conventional high-temperature production of electrolytic aluminum as represented by the Hall-Héroult method.

To further reduce the energy consumption of aluminum production, it is necessary to develop a low-temperature (≤300°C) approach to producing electrolytic aluminum technology, utilizing novel electrolytes to achieve this. In recent years, the emergence of ionic liquids has provided an opportunity to obtain suitable electrolytes for the low-temperature production of electrolytic aluminum.

## 3 Low-temperature ionic liquid-based electrolytic aluminum deposition

### 3.1 Classification of ionic liquids

Ionic liquids composed of positive and negative ions are in a liquid state at RT and are also known as RT molten salts. Usually, ionic liquids can maintain a liquid state at a wide range of temperatures from −90 to 300°C. Although there is currently no clear distinction between ionic liquids and molten salts, salts with a melting point lower than 100°C can be classified as ionic liquids, while others are considered as common molten salts.

**FIGURE 3:**
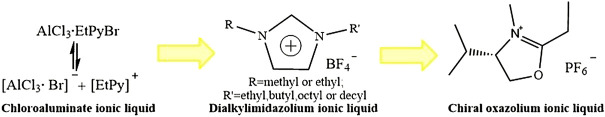
Schematic view of the three generations of ionic liquids.

In 1914, Walden et al. reported the first ionic liquid, ethylamine nitrate, but this ionic liquid was unstable and prone to combustion (Walden, 1914). In 1948, Hurley and Wier heated and mixed aluminum trichloride and halogenated ethylpyridine to obtain a colorless transparent liquid. This was the first generation of ionic liquids known as chloroaluminate ionic liquids. Later in 1992, Wilkes et al. from the United States Air Force Research Institute successfully synthesized water and air stable ionic liquids, such as dialkylimidazole tetrafluoroboric acid and hexafluorophosphoric acid, with good stability and high water resistance that defined the second generation of ionic liquids (Wilkes and Zaworotko, 1992). Since then, more attention has been paid to ionic liquids. In 2002, Wasserscheid et al. synthesized three kinds of ionic liquids containing chiral cations using common chiral materials (Wasserscheid et al., 2002) and established the third generation of ionic liquids, namely functionalized ionic liquids. These ionic liquids with chiral or other special features were also known as “designer solvents”, synthesized ionic liquids designed to contain the cations or anions needed to meet specific requirements (Chaturvedi, 2011). The evolution path of the generation of ionic liquids can be described in three stages, namely the development of chloroaluminate ionic liquids, dialkylimidazolium ionic liquids, and chiral oxazolium ionic liquids (Figure 3).

As designer solvents, ionic liquids have unique properties that many conventional solvents lack. 1) Wide liquid temperature range. By adjusting the size and structure of the anion and cation, ionic liquids can stay in the liquid state at RT or even lower temperatures, with a liquid temperature range of −90 to 300°C. 2) Strong solvency: Ionic liquids can dissolve many polymers and organic, inorganic, and organometallic compounds with high solubility. 3) Low vapor pressure: The vapor pressure of ionic liquids is almost zero, remaining nearly non-volatile even at high temperatures. 4) Good electrical conductivity: Ionic liquids have excellent electrical conductivity with a wide electrochemical window (up to 4–6 V) and can be used as an electrolyte in electrochemical research. 5) Good thermal stability. Most ionic liquids remain stable at 300°C without any decomposition. In addition, they have good chemical stability and can be stored for extended periods. Considering the abovementioned excellent properties, ionic liquids can be exploited as electrolytes in the preparation of metals by electrolysis, especially those that are difficult to prepare in aqueous solutions, such as aluminum, copper, lithium, and titanium. Specifically, the feasibility study of electrolytic aluminum with ionic liquids has received extensive attention for its potential to reduce the high energy consumption of the Hall–Héroult method.

### 3.2 Differentiating electrolytic aluminum produced by traditional methods and with ionic liquids

Due to the weak oxidation of aluminum, the Hall–Héroult method is currently used for aluminum electrolysis. However, there are many problems with this process, such as its high energy consumption, serious environmental pollution, and excessive greenhouse gas emissions. Electrolytic aluminum deposition using ionic liquids presents the advantage of low energy consumption as a green process.

Some ionic liquids (such as chloroaluminate ionic liquids) can form an electrolyte solution containing a high concentration of Al^3+^ ions at temperatures below 300°C due to their excellent solubility of aluminum compounds (AlCl_3_, *etc.*). This reveals the possibility of reducing the operating temperature of the production of electrolytic aluminum from over 900 to below 300°C. The focus of this approach is to prepare ionic liquids with proper characteristics, including good solubility for Al^3+^ ions, high electrical conductivity, and high thermal and chemical stability. Generally, ionic liquid electrolytes are classified as chloroaluminate or non-chloroaluminate ionic liquids.

## 4 Ionic liquids used in electrolytic aluminum deposition

### 4.1 Chloroaluminate ionic liquids

**FIGURE 4:**
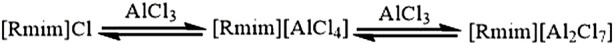
Schematic diagram of electrolyte synthesis using chloroaluminate ionic liquids (Fan et al., 2008).

As the first generation of ionic liquids, chloroaluminate ionic liquids have been widely used as the electrolyte in low-temperature or RT electrolytic aluminum deposition for decades. Many studies using chloroaluminate ionic liquids as the electrolyte have been carried out, including the analysis of nucleation behavior of the aluminum deposition process by cyclic voltammetry, the investigation of the morphology and composition of deposited aluminum by scanning electron microscope (SEM) and X-ray photoelectron spectroscopy, and the examination of the structural stability of deposited aluminum *via* thermogravimetric analysis and scanning tunneling microscopy (Gao et al., 2008; Zhang, 2015). The electrolyte of the chloroaluminate ionic liquids was synthesized by mixing a chloroaluminate ionic liquid with a certain amount of AlCl_3_ (Figure 4).

The Lewis acidity of this ionic liquid can be finely adjusted by changing the molar fraction of AlCl_3_, which is important for aluminum electrodeposition. When *x* (AlCl_3_) < 0.5, where *x* (AlCl_3_) represents the molar fraction of AlCl_3_ in the chloroaluminate ionic liquid, the obtained chloroaluminate ionic liquid is alkaline, and the aluminum exists primarily in the form of AlCl_4_^−^. The obtained chloroaluminate ionic liquid is neutral when *x* (AlCl_3_) = 0.5. Further increasing the molar fraction such that *x* (AlCl_3_) > 0.5 causes AlCl_4_^−^ to further complex with excess AlCl_3_ to form Al_2_Cl_7_^−^, and the acidic chloroaluminate ionic liquid is obtained (Wu et al., 2008). Therefore, a proper electrolyte of the chloroaluminate ionic liquid can be obtained by adjusting the amount of AlCl_3_ added. Compared to the conventional high-temperature Hall–Héroult method, aluminum electrodeposition at lower temperatures (below 100°C) can be achieved in the production of electrolytic aluminum using chloroaluminate ionic liquid. By using an inert electrode material, solid aluminum is precipitated at the cathode and the anode can obtain high-value-added chlorine. The electrochemical reactions at the electrodes and the overall reaction are listed as follows:

$$Cathode:\;4{Al}_{2}{Cl}_{7}^{-}+3e^{-}\;\to \;Al+7{AlCl}_{4}^{-}$$ (1)

$$Anode:\;4{AlCl}_{4}^{-}\;\to \;2{Al}_{2}{Cl}_{7}^{-}+{Cl}_{2}+2e^{-}$$ (2)

$$Overall\;reaction:\;2{Al}_{2}{Cl}_{7}^{-}\;\to \;2Al+3{Cl}_{2}+2{AlCl}_{4}^{-}$$ (3)

The production of electrolytic aluminum using chloroaluminate ionic liquid as an electrolyte has been extensively explored due to its great potential in the commercialization of low-temperature electrolytic aluminum technology. There are many factors, including the reaction conditions, that influence the performance of this method, which are the core of this approach. In this regard, researchers have conducted many related studies and achieved a series of research results.

### 4.2 Non-chloroaluminate ionic liquids

#### 4.2.1 [Tf_2_N] ionic liquids

Many other ionic liquid electrolytes, collectively referred to as non-chloroaluminate ionic liquid electrolytes, were considered in order to address the drawbacks of chloroaluminate ionic liquid electrolytes in the study of electrolytic aluminum. El Abedin et al. reported the electrodeposition of microcrystalline aluminum in an additive-free 1-butyl-1-methyl-pyrrolidine bis(trifluoromethylsulfonimide) ([BMP]Tf_2_N)/AlCl_3_ ionic liquid electrolyte (El Abedin et al., 2005). The microcrystalline aluminum was electrodeposited on the surface of the gold cathode through nucleation growth at RT. A uniform, dense, and bright electrodeposited aluminum layer was acquired with an average grain size below 100 nm. When increasing the operating temperature to above 100°C, nucleation growth disappeared, and a better quality electrodeposited microcrystalline aluminum was obtained with an average grain size of 34 nm. Compared to chloroaluminate ionic liquids, this ionic liquid was easy to purify and dry while also being stable in water and air (water content <1 ppm, 1 mg/L). Furthermore, when the concentration of AlCl_3_ was higher than 1.6 mol/L, the ionic liquid electrolyte would separate into two phases, separating into only one solid phase when the concentration of AlCl_3_ was increased to 2.7 mol/L. However, the biphasic mixture becomes monophasic again when the temperature is 80°C. Subsequently, El Abedin et al. compared the performance of three non-chlorinated acidic ionic liquids, [BMP]Tf_2_N, 1-ethyl-3-methylimidazole bis(trifluoromethylsulfonimide) ([Emim]Tf_2_N), and trihexyl-tetradecyl-phosphoric bis(trifluoromethylsulfonimide) ([P_14,6,6,6_]Tf_2_N) (El Abedin et al., 2006). Bright, nanoscale dense, and well-bonded aluminum was electrodeposited on the cathode in the [BMP]Tf_2_N/AlCl_3_ electrolyte, which was consistent with previous studies. However, only coarse micron-sized cubic crystals of aluminum particles could be obtained in the [Emim]Tf_2_N/AlCl_3_ electrolyte, with the particle size rapidly increasing as the operating temperature increased. In addition, a very thin and mirror-like aluminum deposit formed on the cathode surface in the [P_14,6,6,6_]Tf_2_N-AlCl_3_ electrolyte at RT, and an aluminum deposit with an average crystallite size of 35 nm was produced when the operating temperature was increased to 150°C. In these kinds of ionic liquids, nanocrystalline and mirror-like aluminum deposits can from on the surface of the cathode without applying pulse-plating or adding brighteners. El Abedin et al., Endres et al., and Rüther et al. continued to study [Tf_2_N]-based ionic liquids and further explored the mechanism and influencing factors of aluminum electrodeposition (Moustafa et al., 2007; Rocher et al., 2009; Veder et al., 2013). The different cations and anions, molar ratios of ionic liquids, and Al–Tf_2_N compounds used all influence the behavior of aluminum in AlCl_3_. The experimental results revealed that an appropriate concentration of AlCl_3_ (1.6–2.7 mol/L) was needed when using [Tf_2_N] ionic liquid electrolytes to synthesize electrolytic aluminum at RT (25°C). When the AlCl_3_ concentration was within this range, the electrolyte solution would separate into two phases and the electrodeposition of aluminum would occur only in the AlCl_3_-riched phase. At concentrations above 2.7 mol/L, only a single white solid phase was observed and the electrodeposition of aluminum could not occur in such case.

#### 4.2.2 [N(CN)_2_] ionic liquids

In addition to [Tf_2_N]-based ionic liquid electrolytes, other non-chloroaluminate ionic liquids with a specific anion were explored as the electrolyte in low-temperature electrolytic aluminum deposition. Deng et al. studied [N(CN)_2_]-based ionic liquid electrolytes, including 1-ethyl-3-methylimidazolium dicyanamide (EMI-DCA), 1-butyl-3-methyl-imidazolium dicyanamide (BMI-DCA), and 1-butyl-1-methylpyrrolidine dicyanamide (BMP-DCA), to investigate the electrodeposition characteristics of the metal (Deng et al., 2008). The experimental results indicated that the [N(CN)_2_]-based ionic liquids had lower viscosity and better complexation than the [Tf_2_N]-based ionic liquids, making them an ideal solvent for metal salts. These ionic liquids could be complex with AlCl_3_ or SnCl_2_ to form an electrolyte solution with good water resistance, which is very advantageous for performing electrolysis. It was confirmed experimentally that the low-temperature electrodeposition of aluminum could be achieved using [N(CN)_2_]-based ionic liquid electrolytes. This was a significant breakthrough, since aluminum could only be electrodeposited in chloroaluminate ionic liquids or water and air stable [Tf_2_N]-based ionic liquids prior. [N(CN)_2_]-based ionic liquids expanded the scope of non-chloroaluminate ionic liquid electrolytes viable for low-temperature electrolytic aluminum deposition.

#### 4.2.3 Acetamide ionic liquids

In 2011, Abood et al. (2011) reported a clear, transparent, and yellow ionic liquid that combines acetamide with AlCl_3_ in an equimolar ratio. Characterization based on nuclear magnetic resonance, mass spectrometry, differential scanning calorimetry, and infrared spectroscopy suggested that this ionic liquid remained liquid over a wide temperature range and was insensitive to water. The ionic liquid was formed by the complexation of [AlCl_2_·nAmide]^+^ and AlCl_4_^−^ through the following reaction:

$$2{AlCl}_{3}+nAmide\;⇌\;{\left[{AlCl}_{2}\cdot nAmide\right]}^{+}{AlCl}_{4}^{-}$$ (4)

Low-temperature aluminum electrolysis using the acetamide ionic liquid as the electrolyte was also carried out. A distinct aluminum deposition layer was observed at the cathode after 1 h under a constant current of 2 mA/cm^2^ and a constant voltage of 0.4 V at 25°C. Compared with conventional ionic liquids, the cation of the acetamide ionic liquid includes both the amide-based organic component and the inorganic aluminum metal component. It was the first time that such a unique structure was reported in the cations of ionic liquids, reforming their design and providing a new idea for the synthesis of functional ionic liquids. Based on Abood’s research, Zheng et al. conducted an in-depth systematic study on the electrodeposition of aluminum in acetamide ionic liquid electrolytes (Zheng et al., 2017). Constant current electrolysis was applied to obtain a flat and dense aluminum deposit layer at 303.2–333.3 K and 5–10 mA/cm^2^. The mass purity of aluminum in the deposited layer was above 99%. The results indicated that the production of electrolytic aluminum was controlled by ion diffusion, and the operation parameters (e.g., temperature and current density) had a significant influence on the surface morphology of the aluminum deposition layer. The broad applicability of these ionic liquids is reflected in their use in low-temperature electrolytic aluminum deposition and potential in other similar fields such as electroplating, electrorefining, and alloy manufacturing.

#### 4.2.4 Other types of ionic liquids

Melamed et al. developed the ionic liquid [Hmim][TFSI]/AlCl_3_ for the electrolytic deposition of aluminum, improving the solubility of AlCl_3_ (8 mol/L) and creating a nanocrystalline, single-phase Al face-centered-cubic structure (Melamed et al., 2021). However, in these kinds of ionic liquids, AlCl_3_ is still used as the raw material of aluminum, which increases the operational and production costs. Al_2_O_3_ is presently a more ideal raw material, so its use as a raw material for ionic liquid-based electrolytic aluminum deposition is desirable. Thus far, some researchers have reported their progress in exploring the ionic liquids that might satisfy these requirements. In 2004, Whitehead et al. synthesized a [Bmim]HSO_4_ ionic liquid and initially used it to extract and recover precious metals such as Au and Ag from ore (Whitehead et al., 2004). The extraction yields of Au and Ag from the [Bmim]HSO_4_ ionic liquid were 86% and 60%, respectively. Then, in 2005, Abbott et al. studied the solubility of some metal oxides in this ionic liquid (Abbott et al., 2005). The results revealed that the solubility of Al_2_O_3_ in ionic liquids was low and only at the ppm level. Despite this, researchers continued to advance the progress toward the preparation of Al_2_O_3_-based ionic liquid electrolytes. Besides its low solubility of Al_2_O_3_, [Bmim]HSO_4_ had the merits of its stable physical and chemical properties, low cost, and high cycle utilization (Wang et al., 2009). Wang et al. conducted aluminum electrodeposition in a [Bmim]HSO_4_ ionic liquid electrolyte *via* a potentiostatic method (Wang et al., 2010). The results showed that the electrochemical window of [Bmim]HSO_4_ was as high as 4.83 V, which was wide enough for electrodepositing aluminum in the production of electrolytic aluminum. It was observed that the underpotential electrodeposition of aluminum occurred on the copper electrode. When the current density was 70 mA/cm^2^, a dense and highly adherent aluminum deposit layer was obtained. Ma et al. conducted a feasibility study of Al_2_O_3_ electrolysis in an [Emim]HSO_4_ electrolyte (Ma et al., 2007). The results indicated that [Emim]HSO_4_ was stable at temperatures below 270°C, and the viscosity and electrical conductivity of the electrolyte met the technical requirements. The solubility of Al_2_O_3_ in the [Emim]HSO_4_ ionic liquid (3.81 g/L) was much higher than that in other ionic liquids, meeting the required concentration of Al^3+^ for electrolysis. Mu et al. applied a microwave synthesis method to prepare the [Emim]HSO_4_ ionic liquid and used copper as the electrode material to study aluminum electrolysis (Mu et al., 2012). The experimental results showed that aluminum could be subjected to underpotential and bulk-phase electrodepositions on both platinum and copper electrodes, resulting in uniform aluminum grains and a dense, adherent aluminum layer. The electrolysis of aluminum was diffusion-controlled, and the nucleation mode was between three-dimensional transient nucleation and continuous nucleation.

Existing HSO_4_ ionic liquid electrolytes have the following advantages. 1) They are easily synthesized and insensitive to water and air. 2) The electrolytic aluminum process is simple to operate. 3) These electrolytes show good potential in achieving practical low-temperature electrolytic aluminum production. To differentiate between chloroaluminate and non-chloroaluminate ionic liquids, the properties of different ionic liquids are shown in Table 1.

**TABLE 1:** Performance of different types of ionic liquids.

	Type of ionic liquids	
	Chloroaluminate	Non-chloroaluminate
Stability	Sensitive to water and air	Stable in water and air
State	Liquid	Solid/solid–liquid/liquid
Conductivity	High conductivity (22–23 mS/cm, 303 K) (Zheng et al., 2019)	Low conductivity (0.804 mS/cm, 298.15 K) (Abood et al., 2011)
Solubility of AlCl_3_	>3 mol/L, RT	1.6 mol/L, RT (El Abedin et al., 2005)
System viscosity	Low viscosity (22.54–44.11 mPa s, 293.15 K) (Zheng et al., 2019)	High viscosity (6000 mPa s, 298 K) (Abood et al., 2011)

## 5 Factors affecting the production of electrolytic aluminum

In electrolysis, various factors, such as the electrode material, electrolyte composition, additives, and electrolysis temperature, will affect the final product. To determine an ideal approach to electrolysis, it is necessary to summarize and analyze the factors affecting this process.

### 5.1 Electrode materials

As the substrate in aluminum electrodeposition, the electrodes affect the electrolysis performance of the chloroaluminate ionic liquid electrolyte. Jiang et al. studied the electrodeposition of aluminum using tungsten and aluminum as different cathodes in the ionic liquid electrolyte [Emim]Cl/AlCl_3_ (1:2 M ratio). The current curve showed that the electrodeposition of aluminum on the tungsten cathode was a transient nucleation process of diffusion-controlled growth, while the electrodeposition of aluminum on the aluminum cathode was controlled by kinetics. After constant potential electrodeposition in the potential range of −0.1 to 0.4 V, a continuous, dense, well-attached aluminum deposited layer was obtained on the surface of both cathodes. In addition, the galvanostatic tests showed that when the current density was at 10–70 mA/cm^2^, a dense aluminum deposited layer was obtained on the aluminum cathode, achieving a Faraday efficiency of 85–100%. The Faraday efficiency, which depends on the current density, decreased when the current density was above 100 mA/cm^2^ with the formation of a rough aluminum deposition layer (Jiang et al., 2006a). Jiang et al. (2006b) also found that the kind of ionic liquid electrolyte influences the electrodeposition of aluminum on the cathode. For instance, when trimethyl-phenyl-ammonium chloride (TMPAC)-AlCl_3_ was used as the electrolyte (1:2 M ratio), the electrodeposition of aluminum on the aluminum cathode became an instantaneous diffusion-controlled nucleation process. It was also suggested that the tungsten cathode could cause the partial electrodeposition of aluminum on its surface due to its chemical composition and structure. This also confirmed the importance of electrode materials in the aluminum electrodeposition process. In addition to pure metal cathodes (e.g., tungsten and aluminum), the electrodeposition of aluminum using alloy electrodes, including Li–Al and Nd–Fe–B alloys, was also reported. Bardi et al. (2009) reported aluminum electrodeposition on a P90 Li–Al alloy using [Bmim]Cl/AlCl_3_ at RT. On the surface of the polished P90 Li–Al alloy, a uniform and dense aluminum layer was obtained at an electrodeposition rate of 10 μm/h. Jiang et al. studied aluminum electrodeposition on the surface of the Nd–Fe–B alloy using [Emim]Cl/AlCl_3_ (1:2 M ratio) (Jiang et al., 2016). The surface morphology, elemental compositions, and substrate bonding of the aluminum layer were respectively analyzed *via* SEM, energy dispersive spectroscopy (EDS), and scribe stripping. The experimental results indicated that aluminum was electrodeposited on the surface of the Nd–Fe–B alloy, forming a dense and continuous layer. Liu et al. pre-activated a magnesium alloy before electrodepositing aluminum on its surface using TMPAC/AlCl_3_ ionic liquid as the electrolyte (Quan et al., 2011). The obtained aluminum layer was observed by scanning electron microscopy, and the polarization curve was investigated by cyclic voltammetry. A dense silver-white aluminum layer was obtained, demonstrating that aluminum could be easily electrodeposited on the surface of the magnesium alloy.

### 5.2 Operational parameters

In addition to electrode materials, operational parameters also affect the performance of electrolytic aluminum deposition, influencing the current density, temperature, electrolysis time, electrolyte composition, and even the stirring speed of the aluminum electrolysis reaction. Wang et al. performed aluminum electrodeposition on the surface of low-activity ferritic-martensitic steel in the ionic liquid electrolyte of [Emim]Cl/AlCl_3_ to study the effect of current density (Wang et al., 2016), observing that increasing current density enhanced the adhesion of the aluminum layer to the substrate. The current pulse during electrolysis weakened the concentration polarization and increased the particle density of aluminum on the electrode surface. Moreover, the grain size of electrodeposited aluminum decreased, and the aluminum particles became more spherical as the current density increased. As a result, a smooth and compact aluminum layer with strong substrate bonding and a controllable layer thickness was obtained *via* optimized electrodeposition, in which the current density was controlled between 10 and 20 mA/cm^2^ with proper current pulses every 45–95 min.

Zhang et al. systematically studied the effects of temperature, electrolysis time, electrolyte composition, and reaction stirring speed on ionic liquid-based electrolytic aluminum (Zhang et al., 2016). The ionic liquid of [Bmim]Cl/AlCl_3_ was used as the electrolyte in their studies, and the influence of the above-mentioned parameters on the quality of the electrodeposited aluminum layer was investigated. The results showed that the surface morphology of the aluminum sedimentary layer was more dependent on temperature, with a lower operational temperature (i.e., 318 K) providing a denser and smoother aluminum deposit. The thickness of the electrodeposited aluminum layer showed a linear relationship to the electrolysis time; however, an excessively long electrolysis time would lead to a decrease in current efficiency. The electrolyte composition and the reaction stirring speed had little effect on the morphology of the aluminum deposit. Nevertheless, an appropriate molar ratio of the electrolyte composition ([Bmim]Cl:AlCl_3_ between 1:1.4 and 1:2) and a proper reaction stirring speed (300–500 rpm) would help to obtain a uniform and dense electrodeposited aluminum layer.

### 5.3 Additives

Some researchers reported that adding an appropriate amount of additives (such as organic solvents) to the chloroaluminate ionic liquid electrolyte would improve the quality of the electrodeposited aluminum layer in the production of electrolytic aluminum. Liao et al. concluded that the addition of 45.4 vol% benzene as a co-solvent in the Lewis acidic [Emim]Cl/AlCl_3_ ionic liquid electrolyte (AlCl_3_ molar fraction >50%) significantly improved the quality of the electrodeposited aluminum layer. As a result, a dense aluminum deposit with a thickness of up to 35 μm and an aluminum grain size of 5–10 μm was obtained (Liao et al., 1997). Endres et al. reported that without adding other reagents, the obtained grain size of electrolytic aluminum obtained using a glassy carbon cathode in Lewis acidic [Emim]Cl/AlCl_3_ (AlCl_3_ molar fraction 55%) was larger than 100 nm (Endres et al., 2003). After adding nicotinic acid, the obtained average grain size under the same operation conditions was 14.0 ± 0.3 nm. It was concluded that the additives significantly affected the nucleation and growth of aluminum crystals in the electrolytic aluminum process. Abbott et al. systematically studied the electrolytic aluminum in the [Bmim]Cl/AlCl_3_ ionic liquid electrolyte through multiple techniques, such as NMR spectroscopy, mass spectrometry, cyclic voltammetry, and atomic force microscopy (Abbott et al., 2010). To investigate the effect of additives, toluene and lithium chloride were added to the electrolyte during aluminum electrolysis and the results were analyzed. The introduction of toluene as an additive changed the molar ratios of the aluminum complexes (AlCl_4_^−^ and Al_2_Cl_7_^−^) in the ionic liquid and inhibited the underpotential electrodeposition of aluminum. This affected the morphology of the aluminum deposit and brightened the electrodeposited aluminum layer to a silver-white color. However, the addition of lithium chloride had an adverse effect; it promoted the underpotential electrodeposition of aluminum and produced large aluminum crystallites on the cathode, which darkened the electrodeposited aluminum layer to a dark gray color. Similarly, Bakkar and Neubert. (2015) studied the use of additives. Since chloroaluminate ionic liquid electrolytes are sensitive to water and air, they typically must be used in an inert environment such as a nitrogen-filled glove box, which largely increases the complexity of the electrolytic aluminum production process. To address this, decane, a hydrophobic and low-density organic solvent, was added on top of the chloroaluminate ionic liquid electrolyte as a cover to prevent the electrolyte from contacting water or air. Aluminum electrolysis could then be conducted using a low-carbon steel cathode in the decane-protected [Emim]Cl/AlCl_3_ ionic liquid electrolyte (1:1.5 M ratio). The electrodeposited aluminum layer was examined by SEM and energy-dispersive X-ray spectroscopy. The results indicated that aluminum was uniformly electrodeposited on the cathode surface, achieving a dense and adherent aluminum deposit. The addition of a decane cover prevented the side reaction (i.e., the deterioration) of the chloroaluminate ionic liquid and water absorbed from the air, which not only improved the quality of the electrodeposited aluminum layer but also simplified the production of electrolytic aluminum. This study promoted the development of low-temperature ionic liquid-based electrolytic aluminum, and the effects of various factors on the performance parameters of this process are summarized in Table 2.

**TABLE 2:** Factors affecting the performance of ionic liquid-based electrolytic aluminum ([Emim]Cl/AlCl_3_).

	Factor	Performance
Electrode materials	Aluminum Cathode	Kinetically controlled electrodeposition of aluminum on the aluminum cathode
	Tungsten Cathode	Transient nucleation and diffusion-controlled growth
	P90 Li–Al Alloy	A uniform and dense aluminum layer obtained at an electrodeposition rate of 10 μm/h
	Nd–Fe–B Alloy	A dense and continuous layer obtained on the surface
Operational parameters	Current Density	Enhanced adhesion of the aluminum layer to the substrate with increasing current density
	Electrolyte Composition	Electrolyte composition ([Emim]Cl:AlCl_3_ molar ratio between 1:1.4 and 1:2) would help to obtain a uniform and dense electrodeposited aluminum layer
Additives	Benzene	Significantly improved quality of the electrodeposited aluminum layer
	Nicotinic Acid	Obtained average grain size under the same operation conditions of 14.0 ± 0.3 nm
	Decane	Prevented the electrolyte from contacting water or air

## 6 Applications of ionic liquid-based electrolytic aluminum deposition

Apart from the general production of electrolytic aluminum using ionic liquids, specific applications of this method, including nano-aluminum fabrication, the preparation of aluminum with high purity, and rechargeable aluminum-ion batteries, have been recently investigated. These studies indicated the great potential of ionic liquid-based electrolytic aluminum in the industrial preparation and processing of aluminum and are discussed herein.

### 6.1 Nano-aluminum

**FIGURE 5:**
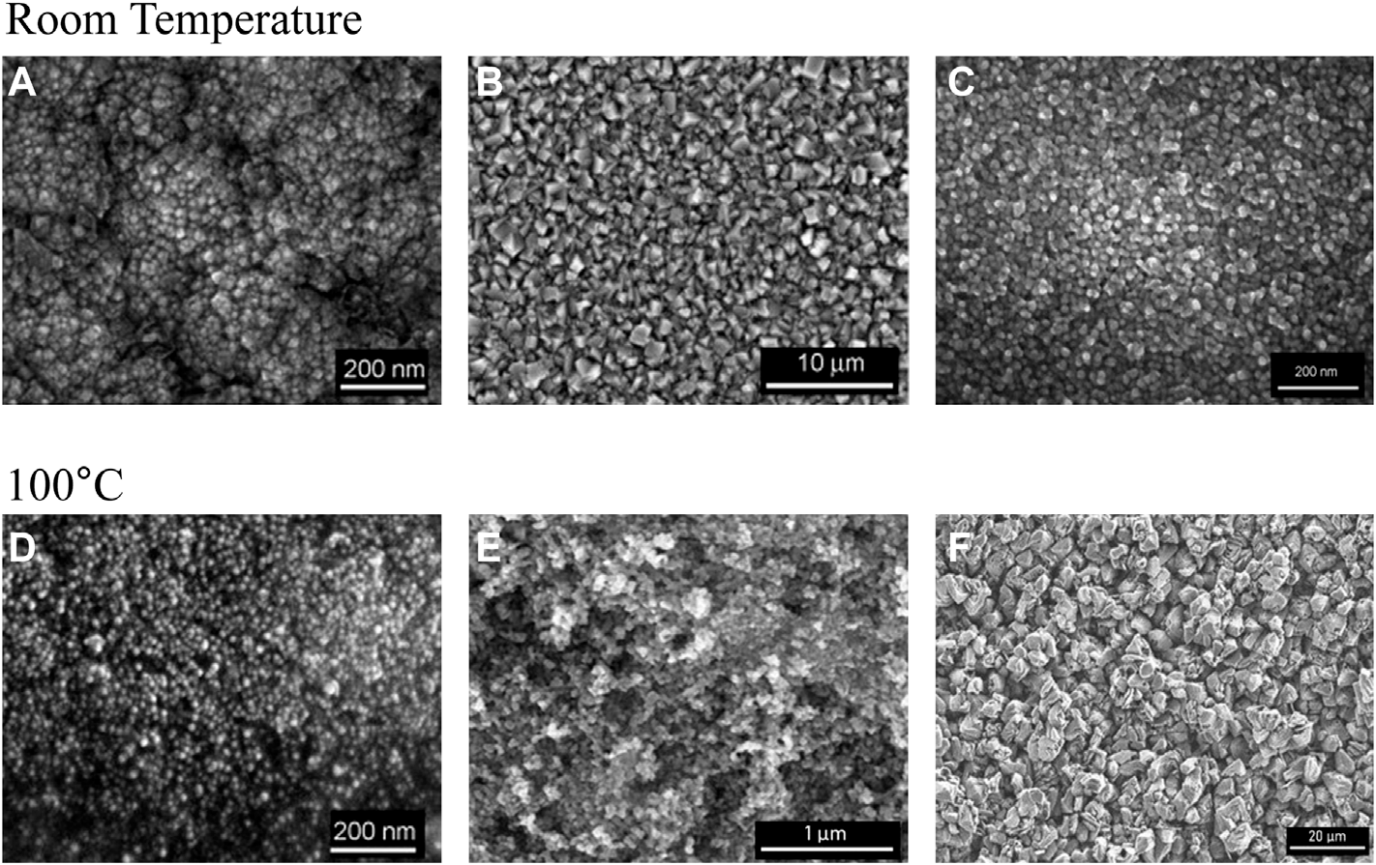
SEM images of nano (micro)-crystalline aluminum obtained from different ionic liquids. **(A)** [BMP][Tf_2_N] Nanoscale. **(B)** [Emim][Tf_2_N]) Microscale. **(C)** [P_14,6,6,6_] [Tf_2_N] Nanoscale. **(D)** [BMP][Tf_2_N] Nanoscale. **(E)** [Py_1,4_][TfO] Nanoscale. **(F)** [Emim][TfO] Microscale.

Nano-aluminum is an advanced material that has a high surface area and robust chemical activity due to its ultra-small particle size (10–100 nm). When exposed to air, nano-aluminum oxidizes vigorously and releases large amounts of heat. Nano-aluminum can be used to significantly increase the specific impulse of rocket propellant, improve the resistance of fuel agglomeration, and enhance fuel ignitability. Therefore, nano-aluminum is widely used in industrial fields such as astronautics, the military, and electronics (Ivanov et al., 2003). At present, the practical methods of fabricating nano-aluminum are primarily physical methods using traditional processing techniques, e.g., evaporation and condensation. Although these physical methods are simple in operation, they are hindered by their high operating temperature, high energy consumption, unsafe manufacturing process, and high risk of explosion, which limits the advancement of nano-aluminum production and its application. Therefore, a greener, safer, and more efficient method to fabricate nano-aluminum is highly needed. Benefitting from the unique structure and good electrochemical properties of ionic liquids, the preparation of nano-aluminum using ionic liquid-based electrolytic aluminum has become a research hotspot, avoiding the problems of conventional physical methods while also improving the quality of the prepared nano-aluminum. In 2003, Enders et al. first reported a nano-aluminum with an average particle size of 14 nm using [Emim]Cl/AlCl_3_ as the electrolyte and nicotinic acid as the organic additive (Endres et al., 2003). The nano-aluminum grains would aggregate to form aluminum crystals, and the average diameter of those crystals exceeded 100 nm if nicotinic acid was not added. These results indicated that electrodeposition nano-aluminum crystal was smoother if proper additives were added to the electrolyte solution. In addition, some pyridyl compounds were also used as additives in the preparation of nano-aluminum *via* ionic liquid-based electrolytic aluminum deposition (Liu et al., 2012; Wang et al., 2015). For instance, the proper addition of nicotinamide in the ionic liquid electrolyte resulted in a highly uniform and smooth electrodeposited nano-aluminum crystal with a size of 14–30 nm (Zhang et al., 2014), which indicated the significance of additives in the formation of nano-aluminum. However, additives were not always needed in the preparation of nano-aluminum *via* ionic liquid-based electrolytic aluminum deposition. In 2005, El Abedin et al. (2005) prepared nano-aluminum with an average size of 34 nm in a [BmPy][Tf_2_N]/AlCl_3_ electrolyte without any additives. The results showed that nano-aluminum was electrodeposited on the Au electrode through a nucleation growth process at RT. When the operating temperature reached 100°C, the minimum particle size of nano-aluminum was 20 nm, and a dense, uniform, and bright electrodeposited nano-aluminum layer was observed. In addition, they nano-aluminum fabricated using three kinds of ionic liquids, [BmPy][Tf_2_N], [Emim][Tf_2_N], and [P_14,6,6,6_][Tf_2_N] (El Abedin et al., 2006), successfully preparing nano-aluminum in each case. In addition, Giridhar et al. found that the 1-butyl-1-methyl pyrrolidine trifluoro-methane sulfonate ([BmPy][TfO]) ionic liquid could also be used as the electrolyte in the preparation of nano-aluminum (Giridhar et al., 2012a). The experimental results showed that nano-aluminum deposits with a particle size of 40–50 nm could be obtained at 100°C when the aluminum chloride concentration was 2.75 mol/L. If the ionic liquid electrolyte was replaced by [Emim][TfO], only micro-sized aluminum crystallite structures formed. These results suggested that the specific cations and anions used in ionic liquid electrolytes could influence the preparation of nano-aluminum in ionic liquid-based electrolytic aluminum deposition (Figure 5). For example, owing to their good stability in humid environments, trifluoro-methyl anions can be used with conventional chloride anion-based ionic liquid electrolytes to prepare nano-aluminum. The potential influence of the structures of the cations was also investigated. Eiden et al. (2009) studied the composition morphology of AlCl_3_ through both experimentation and theoretical calculations for [BMP][Tf_2_N] and [Emim][Tf_2_N] ionic liquids. The types and structures of the cations in the ionic liquid significantly affected the morphologies of the aluminum deposits. In 2012, Giridhar et al. (2012) synthesized nano-aluminum crystals by electrodeposition, using a mixture of [Py_1,4_]Cl/AlCl_3_ and [Emim]Cl/AlCl_3_ ionic liquids (volume ratio no lower than 4:1) as the electrolyte. The results indicated that micro-sized aluminum crystalline structures formed when the volume fraction of [Emim]Cl/AlCl_3_ exceeded 20%.

However, the above studies do not provide a normative and systematic explanation for the relationship between the structure of cations in the ionic liquid electrolyte and the formation of nano-aluminum. To further investigate this relationship, Atkin et al. (2009) studied the interaction between the ionic liquid electrolyte (e.g., [Emim][Tf_2_N] and [BmPy][Tf_2_N]) and the Au (111) substrate using multiple techniques such as atomic force microscopy and scanning tunneling microscopy. Compared to [Emim]^+^, the electric charge of [BmPy]^+^ was more concentrated, which contributed to its stronger cationic surface effect. This resulted in the stronger adsorption of [BmPy]^+^ on the surfaces of the produced aluminum nucleus and Au 111) substrate, preventing the aluminum crystal grains from undergoing continuous growth. Therefore, [BmPy]^+^ was more favorable in the preparation of nano-aluminum deposits. This study emphasized the structure of the imidazole cations, especially the type of side chain, which would significantly influence the preparation of nano-aluminum in imidazole-based ionic liquids. Hereafter, researchers focused on the effect of the side chain in imidazole-based ionic liquids on the nano-aluminum fabrication process (El Abedin et al., 2010; Ismail, 2016). The results revealed that a methoxy group in the side chain of the imidazole cation allowed for the formation of 40 nm nano-aluminum grains without any additives. If an aromatic group was introduced to the imidazole cation, small and thin rod-like aluminum nanocrystalline structures were obtained. These experimental studies revealed that adjusting the functional groups of the ionic liquid electrolyte could significantly alter the dynamic process of the formation of the aluminum nucleus on the electrode surface and the kinetics of the grain growth of the aluminum crystal, which is important in the successful preparation of fine and uniform nano-aluminum particles. Through systematic experiments and theoretical calculations, Zheng et al. (2016) initially found that introducing a Lewis alkaline double-bond structure to the side chain group of the imidazole cation would weaken the interactions between cations and anions, which aided in the formation of a nano-aluminum electrodeposition layer. The strength of the interactions between cations and anions depended on the side chain structure of the ionic liquids, specifically the functional groups in that structure. As the energy barrier of the interactions between cations and anions decreased due to the presence of the Lewis alkaline double-bond structure in the imidazole cation, [Al_2_Cl_7_]^−^ anions in the chloroaluminate ionic liquid electrolyte readily passed through the electric double layer on the surface of the electrodes. This led to a higher density of aluminum nucleation and prevented the formation of large-sized aluminum crystals. Based on the results of Zheng’s research, Wang et al. (2017) utilized SEM, X-ray diffraction, elemental analysis, and Raman spectroscopy as well as density functional theory calculations to study the relationship between the cation structure of the ionic liquid electrolytes and the quality of the electrodeposited nano-aluminum layer. This study further consummated Zheng’s conclusion and revealed the importance of the cation structure of the ionic liquid electrolyte in the preparation of nano-aluminum, which laid a theoretical foundation for subsequent nano-aluminum fabrication experiments and provided a future research direction.

### 6.2 High-purity aluminum

As an indispensable raw material, high-purity aluminum is vital to the fabrication of various advanced materials and elements, including super alloys, integrated circuit wiring, optoelectronic storage media, and many other applications. Due to its excellent properties, high-purity aluminum has been widely applied in aeronautics and astronautics, precise instruments, electronic information devices, and other innovative and high-technology fields. In the industrial preparation of high-purity aluminum, primary aluminum is refined by a three-layer electrolytic process combined with segregation to obtain high-purity aluminum (>99.9% purity). Though the three-layer electrolytic refinement of aluminum was developed at an earlier time and is suitable for industrial production, it still requires high temperatures (above 700°C) that lead to high energy consumption. The amount of energy needed per kilo of high-purity aluminum is as high as 12–18 kW h. Moreover, the segregation process is limited by its low production efficiency. Therefore, the emergence of ionic liquid-based electrolytic aluminum deposition at low temperatures is quite meaningful to improve the production efficiency and reduce the energy consumption of the preparation process. To prepare high-purity aluminum by ionic liquid-based electrolytic aluminum deposition, primary aluminum (about 99% purity) is used as the anode, which is immersed in the ionic liquid electrolyte. The refinement of aluminum is achieved *via* an electrolytic reaction at lower temperatures (25–80°C), and high-purity aluminum is produced on the cathode. The energy consumption of this method is much lower than that of three-layer aluminum electrolysis. Therefore, the preparation of high-purity aluminum *via* ionic liquid-based electrolytic aluminum deposition is a more environmental-friendly approach. It also allows for the recycling of aluminum from the aluminum alloy and aluminum-based composites.

Thus far, many researchers have reported on the preparation of high-purity aluminum using ionic liquid-based electrolytic aluminum deposition. Usually, chloroaluminate ionic liquid electrolytes are used in such studies. The electrochemical reactions involved in this approach are as follows (Zhong et al., 2014):

$$Anodic\;reaction:\;Al+7{AlCl}_{4}^{-}\;\to \;4{Al}_{2}{Cl}_{7}^{-}+3e^{-}$$ (5)

$$Cathodic\;reaction:\;4{Al}_{2}{Cl}_{7}^{-}+3e^{-}\;\to \;7{AlCl}_{4}^{-}+Al$$ (6)

Kamavaram et al. performed a series of studies focusing on the electrolysis and refinement of the aluminum alloy in the [Bmim]Cl/AlCl_3_ ionic liquid electrolyte (Kamavaram et al., 2002; Kamavaram et al., 2003; Kamavaram et al., 2005). Experimental results suggested that high-purity aluminum (>99.89% purity) was obtained by the electrolysis and refinement of aluminum alloys (A358, A360, and A380) in the acidic [Bmim]Cl/AlCl_3_ electrolyte, demonstrating the feasibility of ionic liquid electrolytes in the preparation of high-purity aluminum. In addition, it was concluded that a high operating voltage (1.8 V) and a medium electrolyte concentration ratio ([Bmim]Cl:AlCl_3_ = 1:1.5–1.8) would provide the optimal conditions for the electrodeposition process. The energy consumption of production of high-purity aluminum *via* ionic liquid-based electrolytic aluminum deposition would decrease to a minimum of 3.0 kW h/kg Al, which is much lower than that of conventional methods (12–18 kW h/kg Al), greatly reducing the environmental impact of the process. Reddy et al. studied the preparation of high-purity aluminum by the electrolysis and refinement of aluminum alloy in [C_6_mim]Cl/AlCl_3_ and [C_4_mim]Cl/AlCl_3_ ionic liquid electrolytes, respectively (Wu et al., 2000; Zhang et al., 2003; Zhang et al., 2005; Wu et al., 2016). The results indicated that the current efficiency could reach as high as 99% at an operating voltage of 2.7–3.4 V and a current density of 200–700 A/m^2^. As a result, high-purity aluminum (>99.9% purity) was electrodeposited on the cathode with a thickness of 0.1–0.2 mm. El Abedin (2012) also studied the electrolytic refinement of two alloys, Al–3%Cu and Al–6%Si, in [Emim]Cl/AlCl_3_ and [Bmim]Cl/AlCl_3_ ionic liquid electrolytes to investigate the electrochemical behaviors of both alloys. The results showed that the alloys behaved similarly in both ionic liquid electrolytes, with the dissolution efficiency and current efficiency of each reaching almost 100%. The energy consumption using Al–Cu and Al–Si alloys to refine aluminum in the ionic liquid electrolyte was about 2 kW h/kg Al, which was much lower than conventional refining methods used in the industry. This research not only revealed the potential of chloroaluminate ionic liquid electrolytes in the preparation of high-purity aluminum through electrolytic refinement but also provided a theoretical basis for the preparation of high-purity aluminum using aluminum alloys containing high amounts of metal impurities (Si, Cu, *etc.*).

Given Abedin’s research, Pei et al. (2012b) focused on these impurities (e.g., Zn, Fe, and Cu) and conducted systematic studies on the influence of impurities on the preparation of high-purity aluminum in the [Bmim]Cl/AlCl_3_ ionic liquid electrolyte. The results suggested that most miscellaneous metal elements in the aluminum alloy anode could be removed under the operating conditions of 100 A/m^2^ and 80°C, and a dense and flat electrodeposited aluminum layer was observed on the cathode. The current efficiency exceeded 94% and the energy consumption was 1.59–1.74 kW h/kg-Al. As a result, aluminum with 99.9% purity was produced from the electrolytic refinement of the 75.3% purity industrial aluminum alloy. Pradhan et al. (2009) explored the influence of the surface modification of the cathode on aluminum refinement *via* electrolysis using the [Emim]Cl/AlCl_3_ (1:1.65 M ratio) ionic liquid electrolyte. The results suggested that the cathodic overpotential could be reduced by modifying the cathode surface, suppressing the generation of dendritic aluminum on the cathode surface. Combining this experimental data with theoretical calculations, we concluded that the cathodic overpotential should be lower than −0.55 V to prevent the formation of dendrite-shaped aluminum sediments. A systematic study of the stirring speed, operating temperature, AlCl_3_ concentration in the ionic liquid electrolyte, electrodeposition time, and other conditions was then conducted to investigate their effects on the production of high-purity aluminum with dendrites *via* ionic liquid-based electrolytic refinement (Pradhan and Reddy, 2012). The results revealed that in addition to modifying the electrode surface and controlling the cathodic overpotential, the stirring speed also played an important role in this process.

The use of additives can also impact the preparation of high-purity aluminum through the ionic liquid-based electrolytic refinement of aluminum. Zhao. (2007) studied the influence of two additives, toluene and ammonium chloride, on the electrolytic refinement of aluminum using [Bmim]Cl/AlCl_3_ as the electrolyte. Adding ammonium chloride caused the aluminum dendrites to become thin and bright on the surface of the electrodeposited aluminum layer, while adding toluene darkened the aluminum dendrites formed. Afterward, Li et al. carried out comprehensive studies on the mechanisms of how different additives influenced the electrolytic refinement of aluminum using [Bmim]Cl/AlCl_3_ ionic liquid as the electrolyte (Li et al., 2010; Li et al., 2011b; Li et al., 2011c; Pei et al., 2012a). It was concluded that introducing additives can alter the nucleation mechanism of aluminum on the cathode, significantly reducing the tank voltage, improving the current efficiency, and proving high-quality electrodeposition. When an aluminum alloy of 90% purity was used as the anode, a low energy consumption of 1 kW h/kg Al was obtained and high-purity aluminum (99.9% purity) was produced through the electrolytic refining process, consuming 17% less energy compared to the same process without additives. Wang and Liu (2011) investigated the influence of sodium chloride (NaCl) as an additive on the electrolytic refinement of aluminum using [Bmim]Cl/AlCl_3_ as the electrolyte. Adding NaCl was found to increase the current efficiency to 95.4% and improve the quality of the electrodeposited aluminum layer, decreasing the grain size of aluminum crystalline structures and making the grains brighter. The purity of aluminum in the electrodeposition layer of the cathode could exceed 99.9% without any detectable miscellaneous elements from the anode, fulfilling the goal of the electrolytic refinement of aluminum.

Zhang. (2011) discussed the mechanism of the electrolytic refinement of waste aluminum (6063 aluminum alloy) in an AlCl_3_–NaCl system and the influence of different operating conditions on the electrolytic refinement of the aluminum alloy. The study revealed that the electrodeposition of aluminum on the cathode was a three-dimensional instantaneous nucleation process. A low electrodeposition potential, high operating temperature, and high concentration were conducive to the formation of a crystal nucleus, thereby increasing the rate of nucleation and making the electrodeposited aluminum layer compact and dense. Trace amounts of Si, Fe, Cu, and other miscellaneous elements were detected on the cathode surface, while Mg was dissolved in the electrolyte solution, resulting in a high aluminum purity of over 99.8% at the cathode.

Significant progress in the production of high-purity aluminum *via* ionic liquid-based electrolytic aluminum deposition was realized through the modification of reaction conditions, separation of impurities, and purification of the production process. Furthermore, the utilization of ionic liquid electrolytes can significantly reduce the operating temperature, decreasing the energy consumed in the refining process, simplifying the experimental conditions, and achieving an environmentally friendly chemical process. Though this approach showed good experimental results and lays a solid foundation for further studies to improve the energy efficiency and quality of aluminum production, the studies are still in the experimental stage. No pilot-stage testing or practical ionic liquid-based electrolytic strategies aimed at the production of high-purity aluminum have been reported thus far.

### 6.3 Rechargeable aluminum ion batteries

Compared with lithium, aluminum has the advantages of low cost, high stability, and high charge storage capacity, but the system can withstand fewer cycles (<100 cycles) and suffers from rapid capacity decay. In the past decades, researchers have been committed to solving these problems to achieve the commercial application of aluminum ion batteries. Lin et al. (2015) used three-dimensional graphite as the cathode material and an ionic liquid as the electrolyte to produce an aluminum ion battery with a high cycle number (up to 7500 cycles), high energy density (3 kW/kg), and fast charging ability. However, the chloroaluminate ionic liquid used in the system is sensitive to water and air and poses safety risks in its processing and use. To address the sensitivity of the chloroaluminate ionic liquid to air and water, Sun et al. (2016) used the ionic liquid to prepare gel electrolytes for the aluminum-ion batteries. This system showed good stability in water and air, providing aluminum ion batteries with better mechanical stability. Shen et al. further discussed the current problems encountered in the application of chloroaluminate ionic liquid in aluminum-ion batteries and described the mechanism of the use of an ionic liquid electrolyte in the charging and discharging of aluminum-ion batteries. At the same time, liquid metal was used to improve the performance of the aluminum-ion batteries (Shen et al., 2021). Compared with other kinds of aluminum-ion batteries, the batteries utilizing ionic liquids also showed good performance. Ma et al. (2021) summarized the application of different electrolyte systems in aluminum-ion batteries in detail, noting the necessity of exploring different kinds of ionic liquid electrolyte systems and concluding that the use of ionic liquid electrolytes is one of the most promising research directions. At present, ionic liquid electrolytic aluminum deposition has been well developed and explored. By exploring its similarities with other ionic liquid-based electrolytic aluminum technology, the optimization of aluminum ion batteries with high stability can be achieved to realize the commercial application of rechargeable aluminum ion batteries for large-scale energy storage.

## 7 Current problems with ionic liquid-based electrolytic aluminum deposition

### 7.1 Ionic liquids

At present, ionic liquid electrolytic aluminum deposition has been deeply studied and explored in the laboratory, but there are still many issues with the process that need to be addressed. Chloroaluminate ionic liquids are sensitive to water and air, and harsh conditions are required for their synthesis and utilization. Comparatively, non-chloroaluminate ionic liquid electrolytes have lower conductivity, higher liquid viscosity, and lower solubility for the raw materials of aluminum.

### 7.2 In-depth research

Nowadays, research on ionic liquid electrolytic aluminum deposition mainly focuses on the selection of reaction conditions, the characterization of products, and other general exploratory studies. As a result, further research on the electrochemical deposition mechanism of aluminum, the kinetics of the anode/cathode reactions, and the methods to adjust and control the process parameters are currently insufficient. As for the electrodeposition of aluminum, technical problems such as the growth of dendrites, powdery particles, and the thinness of the electrodeposited layers remain unaddressed.

### 7.3 Other fields related to aluminum

The quality of electrolytic aluminum products should be improved, especially for high-value-added products such as nano-aluminum and high-purity aluminum. So far, many studies have focused on the preparation of nano-aluminum and high-purity aluminum, with some products having achieved the industrial requirements of size and purity. However, only lab-scale trials have been completed with no pilot-stage results. Although the development of aluminum-ion batteries has made breakthrough progress, these batteries are still hindered by their insufficient operating voltage, harsh synthesis conditions of the ionic liquid electrolyte, and the corrosion of the metal shell of the battery. It is necessary to further develop suitable positive and negative electrode materials using ionic liquid electrolytes.

### 7.4 Costs and large-scale production

The high cost of synthesizing ionic liquids remains a problem and the large-scale production of ionic liquids has not yet been widely realized, limiting the industrial applications of such advanced materials. Ionic liquids also sue AlCl_3_ as the raw material for electrolysis, which requires an additional unit operation to prepare AlCl_3_, increases the cost and complexity of the whole process, and thus restricts its practical industrial application. Existing electrolytic cells do not satisfy the requirement of the system, necessitating the redesign or modification of suitable electrolytic cells for use in ionic liquid-based electrolytic aluminum systems.

## 8 Discussion

### 8.1 Future development and use of ionic liquid-based electrolytic aluminum deposition

Understanding the structure–activity relationship and reaction process of ionic liquids to establish a more systematic theoretical system of reaction thermodynamics and kinetics. At the same time, it is also important to solve key problems such as the instability of chloroaluminate ionic liquids in air and water as well as their low conductivity, high viscosity, and low solubility for the AlCl_3_ raw material based on the existing research results of chloroaluminate and non-chloroaluminate ionic liquids. Further enhancing the stability and applicability of ionic liquids and their large-scale production is necessary to ensure the reliability and practicability of the ionic liquid electrolytes in aluminum electrolysis.

To optimize the reaction process for the ionic liquid-based electrolysis of aluminum, the following steps are to be taken. A continuous reaction device to produce aluminum products using the ionic liquid electrolyte should be established, allowing the integration and amplification of this technology to be realized. The reaction data of this system is also needed as well as the ideal electrolytic aluminum conditions. The technical research points mainly include the development and operation of the electrolytic cells, the improvement of the anode materials, the stability and optimization of continuous electrolysis reactions, the collection and processing of aluminum products, thorough systematic research to further improve the reaction efficiency of the system, the promotion of raw materials, and the efficient recycling of equipment for the industrial application of ionic liquid-based electrolytic aluminum systems. By deepening research on the application of this technology in aluminum refinement, nano-aluminum production, and aluminum-ion batteries, the industrial application of low-temperature ionic liquid-based electrolytic aluminum deposition can be realized.

### 8.2 Summary and outlook

**FIGURE 6:**
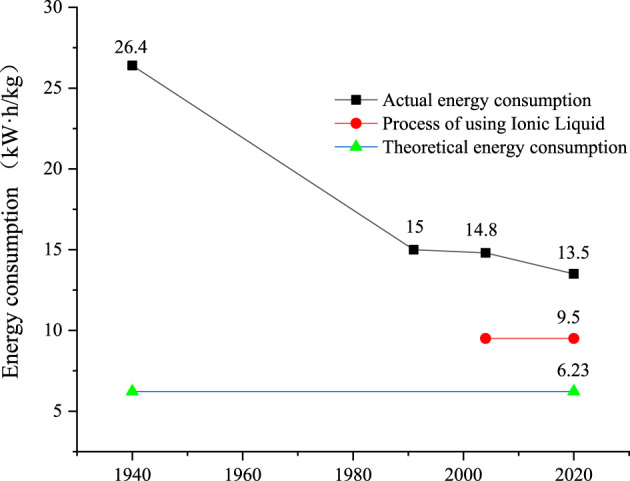
Energy consumption of electrolytic aluminum.

The ionic liquid-based electrolytic aluminum deposition has achieved remarkable development in both experimental and theoretical studies. The pros and cons of this approach have been widely discussed and summarized in this review. The technical and economic indicators of the ionic liquid-based electrolytic aluminum deposition are superior to those of conventional approaches (i.e., the Hall-Héroult method, Table 3). The utilization of ionic liquids in the production of electrolytic aluminum can reduce the emissions of gaseous pollutants (such as CO and CF_4_) and the disposal of solid wastes (such as Al dross), providing a green and environmental-friendly approach (Reddy, 2003). As shown in Figure 6, the energy consumption of the ionic liquid-based approach is close to the theoretical minimum value (6.23 kW h/kg-Al) (Obaidat et al., 2018), saving a great amount of thermal energy compared to the conventional Hall-Héroult method. Moreover, ionic liquid-based electrolysis can also be used to produce high-quality, value-added, and high-purity aluminum for use in rechargeable aluminum ion batteries. The emergence of this promising electrochemical metallurgy technology has provided a new direction for the global electrolytic aluminum industry and promoted its transformation into a green, environmentally friendly, and sustainable industrial model.

**TABLE 3:** Comparison of the technical and economic indices between ionic liquids and the Hall–Héroult method.

Method parameter	Electrolytic aluminum	
	Ionic liquids	Hall-héroult
Tank Voltage (V)	2.0–4.0	4.2–5.0
Energy Consumption (kW·h/t-Al)	9,500–11,000	13,000–19,000
Current Density (mA/cm^2^)	20–100	N/A
Electrode Distance (mm)	5–40	100
Temperature (°C)	25–150	800–1,000
CO Emission (kg/t-Al)	0	340
CO_2_ Emission (kg/t-Al)	0	1,000–1,500
CF_4_ Emission (kg/t-Al)	0	1.5–2.5
HF Emission (kg/t-Al)	0	20–40
Energy Efficiency (%)	>90	<50

## Glossary

[BMP]Tf_2_N: 1-butyl-1-methyl-pyrrolidine bis(trifluoromethylsulfonimide)
[BmPy]Tf_2_N: 1-butyl-3-methylpyridinium bis(trifluoromethylsulfonimide)
BMI-DCA: 1-butyl-3-methyl-imidazolium dicyanamide
BMP-DCA: 1-butyl-1-methylpyrrolidine dicyanamide
[Bmim]HSO_4_: 1-butyl-3-methylimidazolium hydrogen sulfate
[Bmim]Cl: 1-butyl-3-methylimidazolium chloride
[BmPy][TfO]: 1-butyl-1-methyl pyrrolidine trifluoro-methane sulfonate
CF_4_: carbon tetrafluoride
[C_6_mim]Cl: 1-hexyl-3-methylimidazolium chloride
[C_4_mim]Cl: 1-butyl-3-methylimidazolium chloride
CO: carbon monoxide
CO_2_: carbon dioxide
Cu: copper
EDS: energy dispersive spectroscopy
[Emim]Tf_2_N: 1-ethyl-3-methylimidazole bis(trifluoromethylsulfonimide)
EMI-DCA: 1-ethyl-3-methylimidazolium dicyanamide
[Emim]HSO_4_: 1-ethyl-3-methylimidazolium hydrogen sulfate
[Emim]Cl: 1-ethyl-3-methylimidazolium chloride
[Emim][TfO]: 1-ethyl-3-methylimidazolium trifluoro-methane sulfonate
Fe: iron
HF: Hydrofluoric acid
[Hmim][TFSI]: 1-hexyl-3-methylimidazolium bis(trifluormethylsulfonyl)imide
Mg: magnesium
NMR: nuclear magnetic resonance
[N(CN)_2_]^−^: dicyanamide
[P_14,6,6,6_]Tf_2_N: trihexyl-tetradecyl-phosphoric bis(trifluoromethylsulfonimide)
[Py1,4]Cl: 1-butyl-1-methylpyrrolidinium chloride
RT: room temperature
SEM: scanning electron microscope
Si: silicon
Tf_2_N: trifluoromethylsulfonimide
TMPAC: phenyltrimethylammonium
[Py_1,4_] [TfO]: (1-butyl-1-methylpyrrolidinium trifluoro-methane sulfonate)

## References

[B1] Abbott A. P. Capper G. Davies D. L. Rasheed R. K. Shikotra P. (2005). Selective extraction of metals from mixed oxide matrixes using choline-based ionic liquids. Inorg. Chem. 44 (19), 6497–6499. 10.1021/ic0505450 16156600

[B2] Abbott A. P. Qiu F. Abood H. M. Ali M. R. Ryder K. S. (2010). Double layer, diluent and anode effects upon the electrodeposition of aluminium from chloroaluminate based ionic liquids. Phys. Chem. Chem. Phys. 12 (8), 1862–1872. 10.1039/B917351J 20145853

[B3] Abood H. M. Abbott A. P. Ballantyne A. D. Ryder K. S. (2011). Do all ionic liquids need organic cations? Characterisation of [AlCl_2_· nAmide]^+^ AlCl_4_ ^−^ and comparison with imidazolium based systems. Chem. Commun. 47 (12), 3523–3525. 10.1039/c0cc04989a 21301722

[B4] Atkin R. El Abedin S. Z. Hayes R. Gasparotto L. H. Borisenko N. Endres F. (2009). AFM and STM studies on the surface interaction of [BMP] TFSA and [Emim] TFSA ionic liquids with Au (111). J. Phys. Chem. C 113 (30), 13266–13272. 10.1021/jp9026755

[B5] Bakkar A. Neubert V. (2015). A new method for practical electrodeposition of aluminium from ionic liquids. Electrochem. Commun. 51, 113–116. 10.1016/j.elecom.2014.12.012

[B6] Bardi U. Caporali S. Craig M. Giorgetti A. Perissi I. Nicholls J. R. (2009). Electrodeposition of aluminium film on P90 Li–Al alloy as protective coating against corrosion. Surf. Coatings Technol. 203 (10-11), 1373–1378. 10.1016/j.surfcoat.2008.11.003

[B7] Chaturvedi D. (2011). Recent developments on task specific ionic liquids. Curr. Org. Chem. 15, 1236–1248. 10.2174/138527211795202997

[B8] China Industrial Economic Network (2012). New technology of low temperature and low voltage aluminum electrolysis. Available at: http://www.cinic.org.cn/zgzz/cx/136868.html?from=timeline (Accessed January 27, 2021).

[B9] Deng M. J. Chen P. Y. Leong T. I. Sun I. W. Chang J. K. Tsai W. T. (2008). Dicyanamide anion based ionic liquids for electrodeposition of metals. Electrochem. Commun. 10 (2), 213–216. 10.1016/j.elecom.2007.11.026

[B10] Eiden P. Liu Q. El Abedin S. Z. Endres F. Krossing I. (2009). An experimental and theoretical study of the aluminium species present in mixtures of AlCl3 with the ionic liquids [BMP] Tf_2_N and [Emim] Tf_2_N. Chem. Eur. J. 15 (14), 3426–3434. 10.1002/chem.200801616 19229940

[B11] El Abedin S, Z. (2012). Electrochemical behavior of aluminum and some of its alloys in chloroaluminate ionic liquids: Electrolytic extraction and electrorefining. J. Solid State Electrochem. 16 (2), 775–783. 10.1007/s10008-011-1425-5

[B12] El Abedin S. Z. Giridhar P. Schwab P. Endres F. (2010). Electrodeposition of nanocrystalline aluminium from a chloroaluminate ionic liquid. Electrochem. Commun. 12 (8), 1084–1086. 10.1016/j.elecom.2010.05.034

[B13] El Abedin S. Z. Moustafa E. M. Hempelmann R. Natter H. Endres F. (2006). Electrodeposition of nano- and microcrystalline aluminium in three different air and water stable ionic liquids. ChemPhysChem 7, 1535–1543. 10.1002/cphc.200600095 16789040

[B14] El Abedin S. Z. Moustafa E. M. Hempelmann R. Natter H. Endres F. (2005). Additive free electrodeposition of nanocrystalline aluminium in a water and air stable ionic liquid. Electrochem. Commun. 7 (11), 1111–1116. 10.1016/j.elecom.2005.08.010

[B15] Endres F. Bukowski M. Hempelmann R. Natter H. (2003). Electrodeposition of nanocrystalline metals and alloys from ionic liquids. Angew. Chem. Int. Ed. 42 (29), 3428–3430. 10.1002/anie.200350912 12888977

[B16] Fan C. Yan L. Chen Y. Ma J. Li B. (2008). The electro-deposition of aluminum from molten salts at room temperature. Nonferrous Met. Metall. 1, 22–25. 10.1016/S0013-4686(96)00271-X

[B17] Gao L. Wang L. Qi T. Li Y. Chu J. Qu J. (2008). Electrodeposition of aluminum from AlCl_3_/Et_3_NHCl ionic liquids. Acta Physico-Chimica Sin. 24 (6), 939–944. 10.1016/S1872-1508(08)60040-6

[B18] Giridhar P. El Abedin S. Z. Endres F. (2012a). Electrodeposition of aluminium from 1-butyl-1-methylpyrrolidinium chloride/AlCl_3_ and mixtures with 1-ethyl-3-methylimidazolium chloride/AlCl_3_ . Electrochimica acta 70, 210–214. 10.1016/j.electacta.2012.03.056

[B19] Giridhar P. Zein El Abedin S. Endres F. (2012b). Electrodeposition of nanocrystalline aluminium, copper, and copper–aluminium alloys from 1-butyl-1-methylpyrrolidinium trifluoromethylsulfonate ionic liquid. J. Solid State Electrochem. 16 (11), 3487–3497. 10.1007/s10008-012-1800-x

[B20] Hou G. Qiu S. (2011). Benchmarking energy efficiency ‐ the important way for realizing energy saving and emissions reduction of aluminium electrolysis industry. Light Met. 8, 9–11. 10.13662/j.cnki.qjs.2011.08.023

[B21] Ismail A. S. (2016). Nano-sized aluminum coatings from aryl-substituted imidazolium cation based ionic liquid. Egypt. J. Petroleum 25 (4), 525–530. 10.1016/j.ejpe.2015.11.007

[B22] Ivanov F. Osmonoliev M. N. Sedoi V. S. Arkhipov V. A. Bondarchuk S. S. Vorozhtsov A. B. (2003). Productions of ultra‐fine powders and their use in high energetic compositions. Propellants Explos. Pyrotech. 28 (6), 319–333. 10.1002/prep.200300019

[B23] Jiang C. Jin Z. Qi F. Xu K. Xu Y. Zhou J. (2016). Study on preliminary corrosion of NdFeB magnet in ion liquid [AlCl_3_-EMIC]. Electroplat. Pollut. Control 36 (4), 45–48. 10.3969/j.issn.1000-4742.2016.04.017

[B25] Jiang T. Brym M. C. Dubé G. Lasia A. Brisard G. M. (2006a). Electrodeposition of aluminium from ionic liquids: Part I—electrodeposition and surface morphology of aluminium from aluminium chloride (AlCl_3_)–1-ethyl-3-methylimidazolium chloride ([Emim] Cl) ionic liquids. Surf. Coatings Technol. 201 (1-2), 1–9. 10.1016/j.surfcoat.2005.10.046

[B24] Jiang T. Brym M. C. Dubé G. Lasia A. Brisard G. M. (2006b). Electrodeposition of aluminium from ionic liquids: Part II-Studies on the electrodeposition of aluminum from aluminum chloride (AICl3)-trimethylphenylammonium chloride (TMPAC) ionic liquids. Surf. Coatings Technol. 201 (1-2), 10–18. 10.1016/j.surfcoat.2005.12.024

[B26] Kamavaram V. Mantha D. Reddy R. G. (2003). Electrorefining of aluminum alloy in ionic liquids at low temperatures. J. Min. Metall. B. Metall. 39 (1-2), 43–58. 10.2298/jmmb0302043k

[B27] Kamavaram V. Mantha D. Reddy R. G. (2005). Recycling of aluminum metal matrix composite using ionic liquids:. Electrochimica Acta 50 (16-17), 3286–3295. 10.1016/j.electacta.2004.12.002

[B28] Kamavaram V. Reddy R. G. (2002). “July. Recycling of Al-mmc in ionic liquids at near room temperature,” in *ICCE/9, Proceedings of* international *Conference on composites engineering* (LA: David Hui, University of New Orleans), 359–360.

[B29] Li J. Xu Y. Zhang H. Lai Y. (2011a). An inhomogeneous three-phase model for the flow in aluminium reduction cells. Int. J. Multiph. Flow 37 (1), 46–54. 10.1016/j.ijmultiphaseflow.2010.08.009

[B30] Li X. Li J. Lai Y. Liu Y. Zhou H. Cen K. (2006). Influences of gas discharging grooves at bottom of prebaked carbon anodes on bath flow pattern in aluminum reduction cells. Chin. J. Nonferrous Metals 16 (6), 1088–1093. 10.3321/j.issn:004-0609.2006.06.026

[B31] Li Y. Hua Y. Pei Q. Zhang Q. Xu C. Kuang Y. (2011b). Effect of KCl on electrical conductivity of AlCl_3_-BMIC ionic liquid. Light Met. (1), 36–39. 10.13662/j.cnki.qjs.2011.01.009

[B32] Li Y. Hua Y. Zhang Q. Pei Q. Cui Y. Xu C. (2010). Effects of additive choline chloride on electrolysis of aluminum from [bmim]Cl−AlCl_3_ ionic liquid system. Chin. J. Process Eng. 10 (5), 981–986.

[B33] Li Y. Hua Y. Zhang Q. Wang B. Pei Q. (2011c). Effect of tetramethyl ammonium chloride on aluminium electrorefining on copper substrates in AlCl_3_-BMIC ionic liquid system. Light Met. 6, 33–36. 10.13662/j.cnki.qjs.2011.06.022

[B34] Liao Q. Pitner W. R. Stewart G. Hussey C. L. Stafford G. R. (1997). Electrodeposition of aluminum from the aluminum chloride‐1‐methyl‐3‐ethylimidazolium chloride room temperature molten salt + benzene. J. Electrochem. Soc. 144 (3), 936–943. 10.1149/1.1837510

[B35] Lin M. C. Gong M. Lu B. Wu Y. Wang D. Y. Guan M. (2015). An ultrafast rechargeable aluminium-ion battery. Nature 520 (7547), 324–328. 10.1038/nature14340 25849777

[B36] Liu C. (2013). Application of energy saving technology for low- temperature production in traditional aluminum reduction cell. Energy Sav. Nonferrous Metallurgy (4), 121008–145122.

[B37] Liu L. Lu X. Cai Y. Zheng Y. Zhang S. (2012). Influence of additives on the speciation, morphology, and nanocrystallinity of aluminium electrodeposition. Aust. J. Chem. 65 (11), 1523–1528. 10.1071/CH12305

[B38] Ma D. Yuan D. Ponce de León C. Jiang Z. Xia X. Pan J. (2021). Current progress and future perspectives of electrolytes for rechargeable aluminum‐ion batteries. Energy & Environ. Mater. 0, 1–18. 10.1002/eem2.12301

[B39] Ma J. Li Y. Li H. Zhang Y. (2007). Synthesis of 1-Ethyl-3-methylimidazolium hydrogen sulfate and its application in the electrolysis of aluminum. Chin. J. Process Eng. 7 (6), 1083–1088. 10.3321/j.issn:1009-606x.2007.06.006

[B40] Melamed Y. Maity N. Meshi L. Eliaz N. (2021). Electroplating of pure aluminum from [hmim] [TFSI]–AlCl_3_ room-temperature ionic liquid. Coatings 11 (11), 1414. 10.3390/coatings11111414

[B41] Moustafa E. M. El Abedin S. Z. Shkurankov A. Zschippang E. Saad A. Y. Bund A. (2007). Electrodeposition of al in 1-butyl-1-methylpyrrolidinium bis (trifluoromethylsulfonyl) amide and 1-ethyl-3-methylimidazolium bis (trifluoromethylsulfonyl) amide ionic liquids: *In situ* STM and EQCM studies. J. Phys. Chem. B 111 (18), 4693–4704. 10.1021/jp0670687 17388503

[B42] Mu J. Zhang L. Wang E. Lu Y. (2012). Microwave synthesis of [emim] HSO_4_ ionic liquid and its aluminum electrodeposition application. Nonferrous Met. Extr. Metall. 12, 19–22. 10.3969/j.issn.1007-7545.2012.12.006

[B43] National Bureau of Statistics (2022). National date. Available at https://data.stats.gov.cn/easyquery.htm?cn=C01&zb=A0E0H&sj=2021 (Accessed October 05, 2022).

[B44] Obaidat M. Al-Ghandoor A. Phelan P. Villalobos R. Alkhalidi A. (2018). Energy and exergy analyses of different aluminum reduction technologies. Sustainability 10 (4), 1216. 10.3390/su10041216

[B45] Pei Q. Hua Y. Li Y. Gong K. Wang R. Rao S., (2012a). Influences of Zn, Fe and Cu impurities on electrorefining of aluminum in BMIC−AlCl_3_ ionic liquid. Chin. J. Process Eng. 12 (2), 247–252. 10.1007/s11783-011-0280-z

[B46] Pei Q. Hua Y. Li Y. Xu C. Gong K. Wu Z. (2012b). Influence of zinc on electrorefining of aluminium from AlCl3 ‐ BMIC ionic liquid. Light Met. (7), 41–45. 10.13662/j.cnki.qjs.2012.07.018

[B47] Pradhan D. Mantha D. Reddy R. G. (2009). The effect of electrode surface modification and cathode overpotential on deposit characteristics in aluminum electrorefining using EMIC–AlCl_3_ ionic liquid electrolyte. Electrochimica acta 54 (26), 6661–6667. 10.1016/j.electacta.2009.06.059

[B48] Pradhan D. Reddy R. G. (2012). Dendrite-free aluminum electrodeposition from AlCl_3_-1-ethyl-3-methyl-imidazolium chloride ionic liquid electrolytes. Metall. Materi. Trans. B 43 (3), 519–531. 10.1007/s11663-011-9623-1

[B49] Quan L. I. U. Liu K. R. Qing H. A. N. Tu G. F. (2011). Surface pretreatment of Mg alloys prior to Al electroplating in TMPAC-AlCl_3_ ionic liquids. Trans. Nonferrous Metals Soc. China 21 (9), 2111–2116. 10.1016/S1003-6326(11)60981-3

[B50] Reddy R. G. (2003). Emerging technologies in extraction and processing of metals. Metall. Materi. Trans. B 34 (2), 137–152. 10.1007/s11663-003-0001-5

[B51] Ren B. Wang Z. Shi Z. Ban Y. Qiu Z. (2007). Experimental research on anode grooving in large-scale Aluminum electrolytic cell. Min. Metallurgical Eng. 27 (3), 61–63. 10.3969/j.issn.0253-6099.2007.03.015

[B52] Rocher N. M. Izgorodina E. I. Rüther T. Forsyth M. MacFarlane D. R. Rodopoulos T. (2009). Aluminium speciation in 1‐butyl‐1‐methylpyrrolidinium bis (trifluoromethylsulfonyl) amide/AlCl3 mixtures. Chem. Eur. J. 15 (14), 3435–3447. 10.1002/chem.200801641 19132700

[B53] Shen X. Sun T. Yang L. Krasnoslobodtsev A. Sabirianov R. Sealy M. (2021). Ultra-fast charging in aluminum-ion batteries: Electric double layers on active anode. Nat. Commun. 12 (1), 325–328. 10.1038/s41467-021-21108-4 33547316 PMC7864900

[B54] Sun X. G. Fang Y. Jiang X. Yoshii K. Tsuda T. Dai S. (2016). Polymer gel electrolytes for application in aluminum deposition and rechargeable aluminum ion batteries. Chem. Commun. 52 (2), 292–295. 10.1039/C5CC06643C 26511160

[B55] Veder J. P. M. Horne M. D. Rüther T. Bond A. M. Rodopoulos T. (2013). Aluminium oxidation at high anodic potentials in an AlCl3-containing air-and water-stable ionic liquid solution. Electrochem. Commun. 37, 68–70. 10.1016/j.elecom.2013.10.015

[B56] Walden P. (1914). Molecular weights and electrical conductivity of several fused salts. Bull. Acad. Imper. Sci. 1800.

[B57] Wang Q. Zhang Q. Chen B. Lu X. Zhang S. (2015). Electrodeposition of bright Al coatings from 1-butyl-3-methylimidazolium chloroaluminate ionic liquids with specific additives. J. Electrochem. Soc. 162 (8), D320–D324. 10.1149/2.1001507jes

[B58] Wang Q. Zhang Q. Lu X. Zhang S. (2017). Electrodeposition of Al from chloroaluminate ionic liquids with different cations. Ionics 23 (9), 2449–2455. 10.1007/s11581-017-2074-1

[B59] Wang W. Lu G. Zhang G. Yang F. Lai X. (2016). Electroplating aluminum on CLAM steel by chloroaluminate ionic liquids at ambient temperature. Rare Metal Mater. Eng. 45 (5), 1314–1319. 10.1016/S1875-5372(14)60114-4

[B60] Wang X. Liu H. (2011). The effect of NaCl on the electrical conductivity of ionic liquid and recycling of aluminum metal matrix composite. Chemistry 74 (8), 737–741. 10.14159/j.cnki.0441-3776.2011.08.010

[B61] Wang X. Wu W. Tu G. Jiang K. (2009). Synthesis and physical Chemistry properties of acidic 1-butyl-3-methylimidazolium hydrosulfate ionic liquid. Acta Sci. Nat. Univ. Sunyatseni 48 (6), 69–72. 10.3321/j.issn:0529-6579.2009.06.014

[B62] Wang X. Zhang L. Yu X. Dong Y. Li D. Liang H. (2010). Electrodeposition of aluminium from [bmim]HSO_4_ room temperature ionic liquid. Natl. Conf. Phys. Chem. Metallurgy 1, 1000–4343.

[B63] Wasserscheid P. Bösmann A. Bolm C. (2002). Synthesis and properties of ionic liquids derived from the 'chiral pool'. Chem. Commun. (3), 200–201. 10.1039/b109493a 12120366

[B64] Whitehead J. A. Lawrance G. A. McCluskey A. (2004). ‘Green’leaching: Recyclable and selective leaching of gold-bearing ore in an ionic liquid. Green Chem. 6 (7), 313–315. 10.1039/b406148a

[B65] Wilkes J. S. Zaworotko M. J. (1992). Air and water stable 1-ethyl-3-methylimidazolium based ionic liquids. J. Chem. Soc. Chem. Commun. (13), 965–967. 10.1039/C39920000965

[B66] Wu B. Reddy R. G. Rogers R. D. (2000). Aluminum recycling via near room temperature electrolysis in ionic liquids. Recycl. Metals Eng. Mater., 845–856. 10.1002/9781118788073.ch74

[B67] Wu B. Reddy R. G. Rogers R. D. (2016). “Aluminum reduction via near room temperature electrolysis in ionic liquids,” in Essential readings in light metals (Cham: Springer), 1100–1106. 10.1007/978-3-319-48156-2_161

[B68] Wu Q. Han M. Xin H. Dong Q. Jin Y. (2008). Studies on IR spectroscopy and quantum chemical calculation of chloroaluminate ionic liquids acidity. Spectrosc. Spectr. Analysis 28 (2), 282–284. 10.3964/j.issn.1000-0593.2008.02.011 18479004

[B69] Xu W. Li F. (2010). Production practice of reducing dc power consumption of electrolytic aluminum and aluminum liquid is briefly discussed. China Nonferrous Met. (19), 68–70.

[B70] Zhang J. (2011). Studies on waste aluminum alloys Electrorefining in AIC1_3_-NaCI molten salt. Shenyang, China: Doctoral dissertation, Northeastern University.

[B71] Zhang M. Kamavaram V. Reddy R. G. (2005). Aluminum electrowinning in ionic liquids at low temperature. Shenyang, China: Light Metals, 583–588.

[B72] Zhang M. Kamavarum V. Reddy R. G. (2003). New electrolytes for aluminum production: Ionic liquids. Jom 55 (11), 54–57. 10.1007/s11837-003-0211-y

[B73] Zhang Q. Wang Q. Zhang S. Lu X. (2014). Effect of nicotinamide on electrodeposition of Al from aluminium chloride (AlCl_3_)-1-butyl-3-methylimidazolium chloride ([Bmim]Cl) ionic liquids. J. Solid State Electrochem. 18 (1), 257–267. 10.1007/s10008-013-2269-y

[B74] Zhang Q. Wang Q. Zhang S. Lu X. Zhang X. (2016). Electrodeposition in ionic liquids. ChemPhysChem 17 (3), 335–351. 10.1002/cphc.201500713 26530378

[B75] Zhang X. (2015). The preparation of aluminum in ionic liquid and its electrochemical nucleation mechanism *(Master's thesis* . Tianjin, China: Tianjin University). CDMD:2.1016.110183.

[B76] Zhao Q. (2007). Electrorefining of aluminum with BMIC-AlCl_3_-R ionic liquid (doctoral dissertation, kun ming. Kunming, China: Kunming University of Science and Technology.

[B77] Zheng Y. Zheng Y. Dai D. Wang Z. Hou S. (2019). The density, viscosity, and electrical conductivity of three chloroaluminate-based ionic liquids. J. Henan Normal Univ. Nat. Sci. Ed. 47 (6), 65–70. 10.16366/j.cnki.1000-2367.2019.06.010

[B78] Zheng Y. Zheng Y. Peng C. Zhao Z. Tian D. (2016). Electrical double layer in imidazolium chloroaluminate ionic liquids and its influence on the surface morphology of aluminium deposits. Int. J. Electrochem. Sci. 11, 9585–9598. 10.20964/2016.11.88

[B79] Zheng Y. Zheng Y. Wu W. Zhang H. Yan X. (2017). Application of acetamide-based ionic liquid in aluminum electrodeposition. Nonferrous Met. Metall. (9), 15–18. 10.3969/j.sssn.1007-7545.2017.09.005

[B80] Zhong X. Xiong T. Lu J. Shi Z. Hu X. Gao B. (2014). Advances of electro-deposition and aluminum refining of aluminum and aluminum alloy in ionic liquid electrolytes system. Nonferrous Metals Sci. Eng. 5 (2), 44–51. 10.13264/j.cnki.ysjskx.2014.02.008

